# Evaluation of OAR dose sparing and plan robustness of beam-specific PTV in lung cancer IMRT treatment

**DOI:** 10.1186/s13014-020-01686-1

**Published:** 2020-10-17

**Authors:** Yu Chang, Feng Xiao, Hong Quan, Zhiyong Yang

**Affiliations:** 1 Cancer Center, Union Hospital, Tongji Medical College, Huazhong University of Science and Technology, Wuhan, 430022 China; 2 Department of Medical Physics, School of Physics and Technology, Wuhan University, Wuhan, 430072 China

**Keywords:** OAR sparing, Plan robustness, Van Herk’s margin concept, Beam specific PTV

## Abstract

**Purpose:**

Margins are employed in radiotherapy treatment planning to mitigate the dosimetric effects of geometric uncertainties for the clinical target volume (CTV). Here, we proposed a margin concept that takes into consideration the beam direction, thereby generating a beam-specific planning target volume (BSPTV) on a beam entrance view. The total merged BSPTV was considered a target for optimization. We investigated the impact of this novel approach for lung intensity-modulated radiotherapy (IMRT) treatment, and compared the treatment plans generated using BSPTV with general PTV.

**Methods and materials:**

We generated the BSPTV by expanding the CTV perpendicularly to the incident beam direction using the 2D version of van Herk’s margin concept. The BSPTV and general PTV margin were analyzed using digital phantom simulation. Fifteen lung cancer patients were used in the planning study. First, all patient targets were performed with the CTV projection area analysis to select the suitable beam angles. Then, BSPTV was generated according to the selected beam angles. IMRT plans were optimized with the general PTV and BSPTV as the target volumes, respectively. The dosimetry metrics were calculated and evaluated between these two plans. The plan robustness of both plans for setup uncertainties was evaluated using worst-case analysis.

**Results:**

Both general PTV and BSPTV plans satisfied the CTV coverage. In addition, the BSPTV plans improved the sparing of high doses to target-surrounding lung tissues compared to the general PTV plans. Both D_mean_ of Ring PTV and Ring BSPTV were significantly lower in BSPTV plans (38.89 Gy and 39.43 Gy) compared to the general PTV plans (40.27 Gy and 40.68 Gy). The V20, V5, and mean lung dose of the affected lung were significant lower in BSPTV plans (16.20%, 28.75% and 8.93 Gy) compared to general PTV plans (16.69%, 29.22% and 9.18 Gy). In uncertainty scenarios, about 80% of target coverage was achieved for both general PTV and BSPTV plans.

**Conclusions:**

The results suggested that plan robustness can be guaranteed in both the BSPTV and general PTV plans. However, the BSPTV plan spared normal tissues, such as the lungs, significantly better compared to the general PTV plans.

## Introduction

Intensity-modulated radiotherapy (IMRT) can deliver conformal dose distributions to tumors. However, the inter-fractional uncertainties during treatment results in deviations between the planned and actual dose distributions [1–3]. These uncertainties can result in underdosing of the clinical target volume (CTV), or overdosing of organs at risk (OARs) [1].

Current clinical practice accounts for uncertainties by using a safety margin that is defined as the planning target volume (PTV) [4–6]. To achieve a higher probability of the CTV coverage under uncertainties, a larger margin for PTV is needed. However, this can result in a higher dose being delivered to surrounding normal tissues [7]. In the van Herk’s PTV recipe, uncertainties are separated into two types: systematic and random uncertainties [4]. Systematic uncertainties affect all treatment fractions in the same way, but vary stochastically across the patient population. They can be modeled as displacements of the CTV relative to the blurred dose distribution. Random uncertainties vary from treatment to treatment, and can be modeled as blurring of the cumulative dose distribution.

Geometric uncertainties, including both systematic and random uncertainties, might not simply blur the cumulative dose distribution isotropically. In reality, photon beam radiation deposits exponentially attenuation dose with depth, and whilst the lateral fall-off is much sharper [1, 6]. In other words, small displacements in beam direction result in small deviations from the planned dose, whereas displacements perpendicular to beam direction can result in severe underdosing due to the target moving out of the beam penumbra [4]. Therefore, it is physically impossible to generate a dose distribution in photon radiation with a negligible dose outside the PTV due to the low dose bath in the beam direction.

The per-beam margin concept in the proton radiation was first proposed by Peter Park et al. [8]. In their study, this beam specific PTV concept was first used to account for the setup and range uncertainties in the prostate and thoracic sites of the proton radiotherapy. Based on their study, we modified the van Herk’s margin method, generating margins that vary with the beams’ incident directions, thereby only accounting for uncertainties perpendicular to beam incident directions.

Tsang et al. [9] proposed modifications to the van Herk’s margin concept by considering margins on the perpendicular direction of each beam in prostate cancer radiotherapy. Tsang et al. [10] also used an adapted beam dependent margin concept, which combined the beam dependent margin and probabilistic planning optimization together to optimize the trade-off between the target coverage and the surrounding rectum and bladder sparing. Their results showed that using the adapted beam dependent margin, better OAR dose sparing could be achieved compared to the general margin. However, in their study, the plan robustness between the general PTV optimization plan and the beam dependent PTV optimization plan was not compared.

Lung cancer radiation treatment is subject to respiratory and setup uncertainties. The therapeutic ratio in lung cancer is essential to ensure adequate coverage of the moving target volume while sparing surrounding normal tissues. In this study, we focused on generating margins that were dependent on the beams’ incident directions, and merged them as a beam-specific PTV (BSPTV) in lung cancer radiotherapy. Subsequently, we compared the dose distributions between the general PTV and BSPTV optimized IMRT plans, and evaluated the plan robustness for the setup uncertainties between these two optimization strategy plans.

## Materials and methods

### Patient cohort

For this retrospective study, a total of 15 lung cancer patients who underwent IMRT between September 2018 and December 2018 were selected. All patients were enrolled in an institutional review board-approved retrospective data collection protocol, and completed lung radiotherapy treatment.

Patients were acquired 4DCT using a Big Bore CT simulator (Brilliance, Philips Healthcare, Cleveland, OH, USA) with a real-time position management system (RPM, Varian Medical Systems, Palo Alto, CA, USA). The gross tumor volume (GTV) and CTV was contoured on average-weighted CT images. To simplify our analysis, only tumors in one side of lungs were selected. In addition, all OARs, such as lungs and spinal cord were contoured on average-weighted CT images. The average-weighted CT images were used for plan optimization and dose calculation.

### Beam-specific PTV concept

**Fig. 1:**
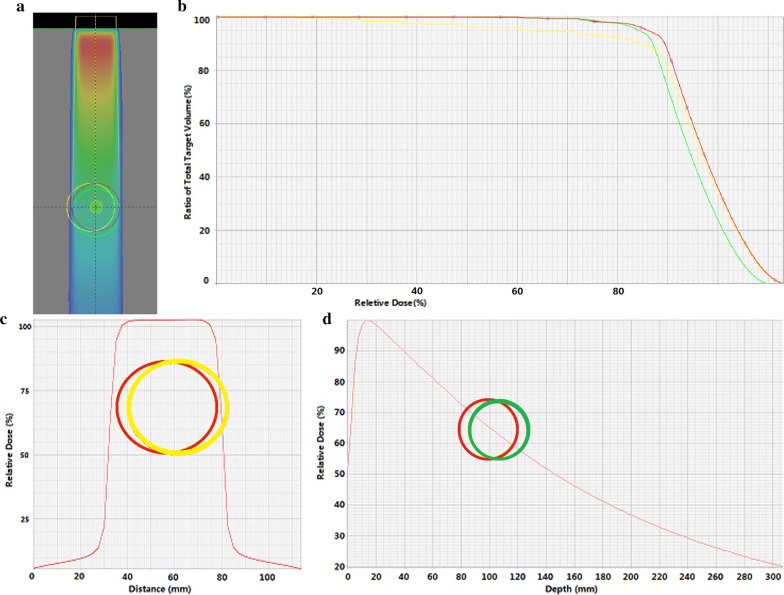
The original target position is indicated by the red circle, **a** target movements for photon irradiation in beam direction (green circle) and perpendicular to beam direction (yellow circle); **b** the DVH of static target (red line) and targets moving in beam direction (green line) and perpendicular to beam direction (yellow line); **c** dose profile between the original target (red circle) and the moved target for photon beam perpendicular to beam direction (yellow circle); **d** percentage depth dose between the original target (red circle) and the moved target for photon beam in the beam direction (green circle)

**Fig. 2:**
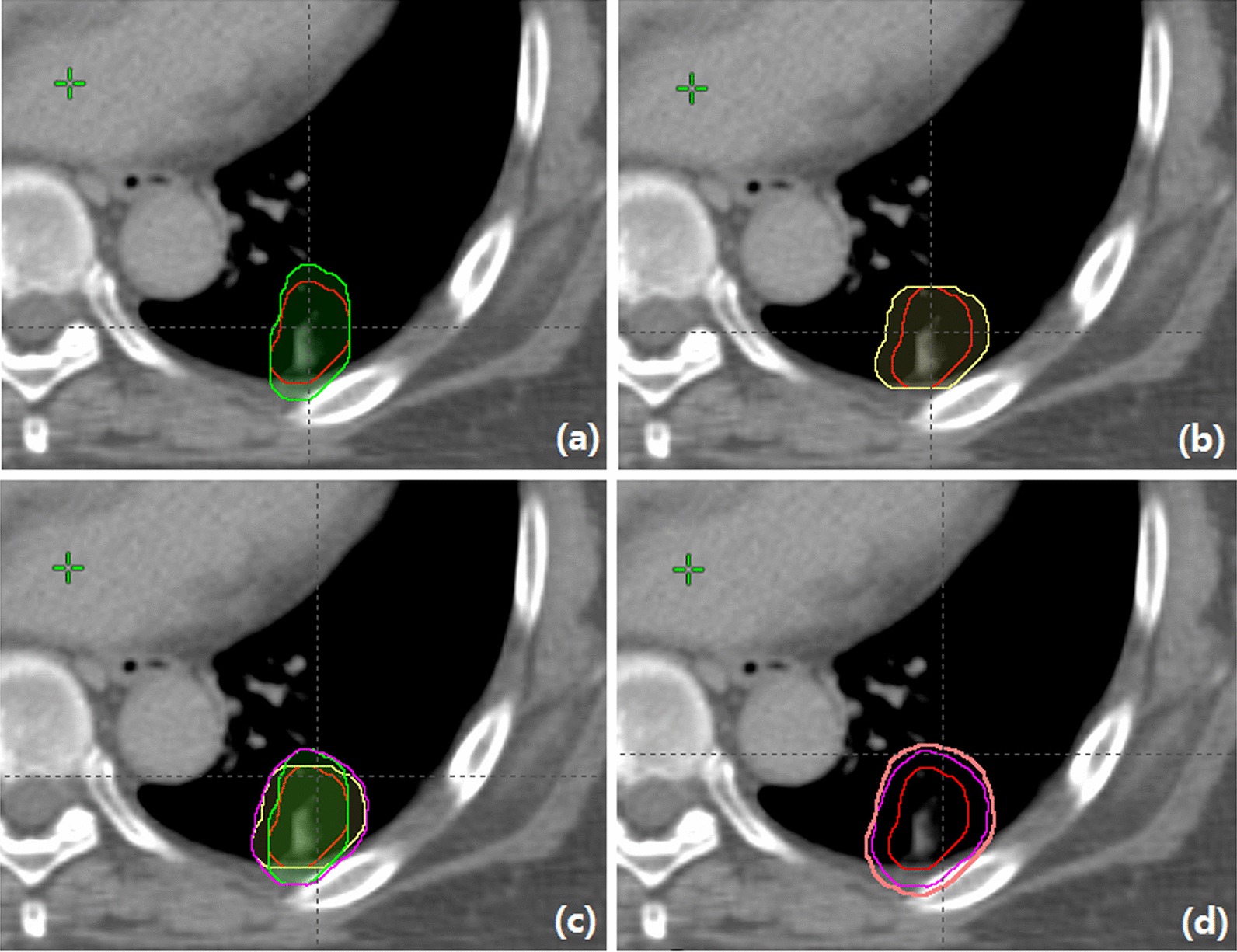
The isocenter axial slice of the CTV (red line) expansion generated using 2D VHMR for 90° beam (green line) in **a** and 180° beam (yellow line) in **b**; **c** shows the isocenter axial slices of the union BSPTV for all beams (pink line); **d** shows the isocenter axial slices of the union BSPTV (pink line) and general PTV (light brown line).

To generate the BSPTV for photon radiotherapy, we expanded the CTV perpendicularly to each incident beam direction using the 2D version of van Herk’s margin concept. We chose not to add margins in the CTV in the incident beam direction, because the percentage depth dose reduction in the incident beam direction was very small. We obtained the final BSPTV by merging each beam expansions. Figure 1 shows the simulation of target coverage differences between the target moving parallel to beam direction and perpendicular to beam direction. Figure 2 shows the geometric differences between the original PTV and the BSPTV for the same CTV in the axial slice.

The setup inter-fractional uncertainties considered in this study were defined in the patient’s left–right, anterior–posterior and superior-inferior directions, and were assumed to be normally distributed with no correlations between them. The projection of the 3D Gaussian distribution, in beam direction, into a 2D Gaussian distribution was defined using the van Herk’s margin concept [4, 9, 11]

$$M = \alpha \Sigma + \beta \sqrt {\sigma^{2} + \sigma_{p}^{2} } - \beta \sigma_{p}$$

Variables Σ, σ, and σ_p_ are two-dimensional column vectors for the directions perpendicular to the incident beam angle, thereby representing the systematic uncertainties, random uncertainties and the beam penumbra, defined as the distance between the 20 and 80% isodose levels, respectively. In this study, the beam penumbras (σ_p_) was 3.2 mm in water. The coefficients α and β, which depend on the intended probability of target dose coverage, were calculated by solving the closed-formed dose population histogram, following the integral formula in Appendix 2 of a previous study [4]. Our method to calculate the 2D margin from the direction perpendicular to each beam was implemented as a standalone MATLAB (MathWorks, Natick, MA, USA) program. To ensure that 90% of the patients received at least 95% of the prescribed dose across the whole of the target, in our programmed 2D margin script, the corresponding coefficients were α = 2.15 and β = 1.64. In addition, we set the systematic uncertainties to Σ = 2 mm, and random uncertainties to σ = 2 mm according to the previous study and our clinical results [4, 12], the Margin (M) ≈ 4.4 mm. The automatic generated 2D margins of each beam were imported into Eclipse TPS (Varian Medical Systems, Palo Alto, CA, USA) and merged as the BSPTV for subsequent optimization.

The general PTV was margined by the 3D van Herk’s margin concept, with the same systematic and random uncertainties as the BSPTV as follows: the systematic uncertainties Σ = 2 mm, random uncertainties σ = 2 mm, α = 2.5, β = 1.64, and M ≈ 5 mm.

### Phantom simulation

A digital water phantom simulation was used to evaluate the conformity of the dose distribution to the OAR sparing of these two margin concepts. The influence of the number of beams to the volume of the BSPTV was also evaluated using this digital water phantom.

In this simulation model, the water phantom was 40 × 40 × 40 cm^3^ with a spherical CTV of 4 cm diameter in the center, which roughly corresponded to the average CTV sizes of our patient data. The dose grid resolution for the dose calculation was 2.5 mm. Two types of 50 Gy/25F treatment plans were designed for both general PTV and BSPTV, and the generated dose distributions were such that 98% of the PTV received 100% of the prescribed dose. The general PTV margin (M ≈ 5 mm) was applied around the CTV for the plans with general PTV as the target volume. The BSPTV margin (M ≈ 4.4 mm, in 0°, 30°, 60° and 90° directions) was applied around the CTV for plans with the BSPTV as a target volume. The first type involved two clinical IMRT plans using 4 coplanar beams with 0°, 30°, 60° and 90° directions with the general PTV and BSPTV as the target volume, respectively. The plan conformity index (CI, CI = 100% × [TV_PI_]^2^/[PI_100_ × TV]) was optimized to keep the CI in both general PTV and BSPTV plans above 80%. TV represented the target volume, TV_PI_ represented the volume of the target covered by the prescribed isodose, and PI_100_ represented the volume receiving 100% of the prescribed isodose. The conformity was better as the index approached 100%. The second type involved hypothetical plans with an ideal dose distribution (such as a VMAT plan with a spherically symmetric dose that falls off in all directions), resulting in a CI above 90%.

Three plans were generated to evaluate the influence of the number of beams to the volume of the BSPTV. The three plans were generated using three, five, or seven coplanar beams with equal angle intervals. Subsequently, the volume difference between the general PTV and BSPTV of the same spherical CTV of each plan was calculated.

### CTV projection area analysis

**Fig. 3:**
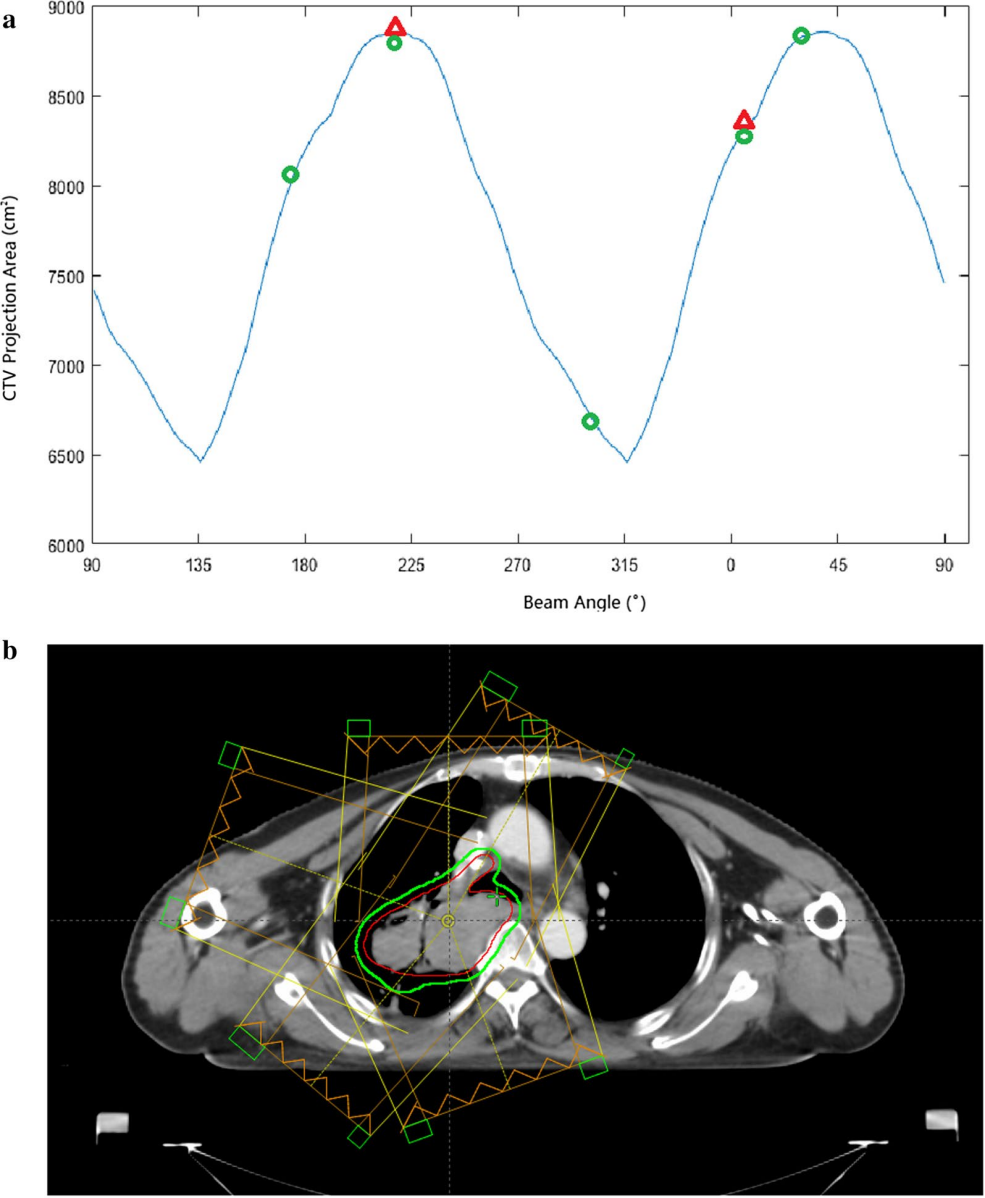
The example case’s CTV projection area with respect to the beam angle is shown in **a**. The selected beam angles (0°, 30°, 160°, 220°, and 290°) are indicated as green circles. The first and second maximum projection beam angles are indicated as red triangles; the axial slice view of the example case’s beams are shown in **b**

In this study, the mathematical relationship between the margin volume and the projection area of the target was analyzed. The calculation and analysis details are shown in “Appendix”. Based on a patient’s CT data set, we calculated the CTV projection area in a beam direction from 0° to 359°. Because the BSPTV of a beam is a 2D margin of the CTV in the beam direction, the projection area of the CTV in the beam direction is an index that can be used to evaluate the volume of 2D expansion of the CTV. The projection area of the CTV was calculated for a full circle of beam angles (0–359) at increments of 1 to yield a patient-specific CTV projection area curve as a function of the beam angle. For example, Fig. 3a shows the CTV projection area of one patient with respect to the beam angle.

### IMRT planning

IMRT plans were optimized by the Eclipse v13.6 treatment planning system (Varian Medical System, Palo Alto, CA, USA) and simulated with 6 MV Xray of Trilogy linac (Varian Medical System, Palo Alto, CA, USA). The plan final dose was calculated using the Acuros algorithm and prescribed to 60 Gy in 30 fractions. First, we selected 4–5 beam angles according to the CTV projection area curve to make sure that the projection area on the YOZ plane had higher values than other beam angles. To avoid the beam penetrating both lungs, most beams incidents were set in the AP directions. Second, the general PTV and BSPTV margins were generated as the description in Beam-specific PTV concept section. Next, two plan optimization strategies were designed to compare the dose distribution differences between the general PTV and BSPTV. The optimization objective for all plans was to first achieve 100% of the prescribed dose to the target volume, then to minimize the dose to OARs. We first optimized all patients’ plans on the target volume of the general PTV. Then, all optimization objective parameters were kept unchanged, a new plan was created, and only the target volume was changed to BSPTV and this BSPTV plan was re-optimized. After the optimization of both plans was completed, all plans were normalized to facilitate dose comparisons. The normalization point was the target D_98%_ of 60 Gy, where D_x%_ was defined as the lowest dose covering x% of the volume. To exclude the influence of the conformity of the dose distribution to the plan evaluation, plans with a CI below 80% were re-optimized until the CI was above 80%.

### Plan evaluation

To quantify the differences between the general PTV and BSPTV plans, dose-volume histograms were used to assess the dose coverage and conformity of targets and the protection of OARs. The target evaluation parameters were D98% (target coverage), D2%, CI, and homogeneity index (HI) [13]. The HI, defined as 100% × (D_2%_ − D_98%_)/D_50%_, was used to evaluate dose homogeneity within each target volume. Plans that are more homogenous, have HI values that are closer to 0% [13]. V5, V20, and mean doses to both lungs were compared. For the spinal cord, the D_1%_ was compared. To evaluation the adjacent normal tissue of CTV, a 2-cm ring of the CTV was margined as the CTV margin. The Ring PTV or Ring BSPTV were created by the CTV margin with the general PTV or BSPTV being subtract. The volume and mean doses of Ring PTV and Ring BSPTV were compared.

### Robust analysis

Over the course of radiation therapy, interfractional uncertainties occur between treatment fractions, such as set-up uncertainties and anatomical variations. In this study, we evaluated the set-up uncertainties using the uncertainty dose evaluation of Eclipse treatment planning system, assuming inter-fractional setup uncertainties of ± 5 mm in AP, LR and SI directions, total 6 scenarios. The van Herk margin recipe was designed for 90% of coverage of the patients with a minimal dose of 95–100% of the target volume, therefore, the ratio of scenarios that satisfied the clinical specification that the 100% of CTV above the 95% prescription dose was evaluated.

### Statistics analysis

SPSS 24.0 software (IBM, Armonk, NY, USA) was used for statistical analyses of all dosimetric metrics. We conducted a paired, two-tailed Wilcoxon signed-rank test to compare the dose distributions between general PTV and BSPTV plans. *P* < 0.05 was considered statistically significant.

## Results

### Phantom simulation

Table 1 shows the phantom simulation results of the 4-field IMRT plans and the hypothetical plans with an ideal dose distribution. For the plans with general PTV set as the target volume, the CI of the 4-field IMRT plans was 85.18% and the CI of the hypothetical plans with an ideal dose distribution was 92.80%. The mean value of the Ring PTV was 53.08 Gy in 4-field IMRT plans, and 53.34 in the hypothetical plans with an ideal dose distribution. For plans with BSPTV set as the target volume, the CI of 4-field IMRT plans was 83.25% and the CI of the hypothetical plans with an ideal dose distribution was 91.92%. The mean value of the Ring PTV was 52.89 Gy in 4-field IMRT plans, and 52.97 in the hypothetical plans with an ideal dose distribution. Both 4-field IMRT plans and hypothetical plans with an ideal dose distribution were 100% satisfied the clinical specification of plan robustness, as shown in Table 2.

**Table 1:** Summary of dose to targets and OARs in water phantom simulation

Parameter	General PTV 4-field IMRT plan	BSPTV 4-field IMRT plan	General PTV ideal dose distribution plan	BSPTV ideal dose distribution plan
CTV, D_98%_	62.5	62.5	61.5	61.0
General PTV, volume (cm^3^)	65.9	65.9	65.9	65.9
General PTV, D_98%_ (Gy)	60.0	59.9	60.0	59.5
General PTV, D_2%_ (Gy)	64.5	64.6	63.3	63.4
General PTV, CI (%)	85.2	85.1	92.8	91.1
General PTV, HI (%)	3.5	3.6	3.4	3.4
BSPTV, volume (cm^3^)	62.5	62.5	62.5	62.5
BSPTV, D_98%_ (Gy)	60.1	60.0	60.2	60.0
BSPTV, D_2%_ (Gy)	64.5	64.6	63.3	63.4
BSPTV, CI	82.4	83.3	90.7	91.9
BSPTV, HI	3.5	3.6	3.4	3.4
Ring PTV, D_mean_ (Gy)	53.1	52.4	53.3	52.6
Ring BSPTV, D_mean_ (Gy)	53.5	52.9	53.7	53.0
Ring PTV, volume (cm^3^)	61.2	61.2	61.2	61.2
Ring BSPTV, volume (cm^3^)	64.6	64.6	64.6	64.6

*HI* homogeneity index, *CI* conformity index

**Table 2:** The ratios of scenarios satisfied the clinical specifications that the 100% target volume being above the 100% or 95% prescription dose in water phantom simulation

Plan name	General PTV plan (%)		BSPTV plan (%)	
	100% prescription dose	95% prescription dose	100% prescription dose	95% prescription dose
4-field IMRT plan	67	100	67	100
Ideal dose distribution plan	67	100	67	100

The volume difference between the general PTV and BSPTV of the same spherical CTV was − 9.06% in the three beams plan, − 7.11% in the five beams plan, and − 5.41% in the seven beams plan.

### CTV projection area analysis

For different patients, the shape of the CTV projection area curve varied. The results of our mathematical and geometrical analysis (“Appendix”) suggested that the smallest BSPTV was obtained with the beam irradiated perpendicular to the maximum average length of the target in the XOY plane. Table 3 lists the beam angles that correspond to the first and second maximum values of the CTV projection area for each patient.

**Table 3:** Volumes for general PTVs and the union of BSPTVs for all patients, and the beam angles corresponding to the extreme values of CTV projection area and the plan selected beam angles

Patient nos.	CTV volume (cm^3^)	General PTV volume (cm^3^)	BSPTV volume (cm^3^)	Volume difference (%)	Beam angles for maximum, 2nd maximum ITV projection area	Selected beam angles
1	36.9	83.9	76.0	− 10.4	204, 180	90, 150, 180, 230
2	11.5	29.2	27.9	− 4.7	175, 210	21, 150, 180, 210, 335
3	6.7	22.9	18.8	− 21.8	300, 320	0, 30, 170, 300
4	87.7	164.5	153.9	− 6.9	102, 90	0, 30, 160, 190, 220
5	12.6	35.0	30.3	− 15.5	90, 315	0, 90, 175, 315
6	3.1	12.9	11.3	− 14.2	195, 180	70, 130, 180, 210
7	270.9	466.2	443.6	− 5.1	0, 220	0, 30, 160, 220, 290
8	10.2	27.6	25.6	− 7.8	106, 320	10, 40, 180, 330
9	1.5	9.0	7.0	− 28.6	160, 125	40, 130, 180, 210
10	57.6	155.4	146.3	− 6.2	45, 90	40, 95, 175, 225
11	12.3	32.9	31.3	− 5.0	0, 210	0, 130, 170, 210
12	8.9	31.1	28.1	− 10.7	130, 145	15, 150, 180, 210, 240
13	72.7	102.8	99.8	− 3.0	320, 50	320, 40, 0, 170
14	9.4	26.0	24.6	− 5.6	270, 240	240, 210, 180, 150, 15
15	14.4	40.0	36.7	− 8.7	250, 180	210, 180, 150, 120, 345

### Volume difference between the general PTV and BSPTV

Beams were selected according to the CTV projection area curve. A larger CTV projection area indicated a smaller 2D expansion of the CTV and more sparing of the adjacent OARs. The beam angles of an example case are presented in Fig. 3b. Table 3 lists the volume of the general PTVs and BSPTVs for all patients. For all patients, the mean (SD) volume reduction of the BSPTV compared to the general PTV was − 10.27% (7.11%).

### Dose difference between the general PTV optimization plan and BSPTV optimization plan

Table 4 shows the general PTV and BSPTV D_98%_, D_2%_, CI, and HI for the two plans. For all patients, the D_2%_ did not significantly differ between the general PTV and BSPTV plans. In addition, the CI of the general PTV in the general PTV optimization plan and the CI of the BSPTV in the BSPTV optimization plan were not significantly different (*p* = 0.281). The CI of the BSPTV was higher in the BSPTV optimization plan compared to the general PTV plan (*p* = 0.003). In both general PTV plans, the D_98%_ of the BSPTV was higher compared to that of the general PTV (*p* = 0.003) and BSPTV plans (*p* = 0.001).

**Table 4:** Summary of dose to targets and OARs for all patients, shown as mean (standard deviation)

Parameter	General PTV optimization plan	BSPTV optimization plan	*P*^a^
CTV, D_98%_ (Gy)	61.68 (0.99)	61.40 (1.15)	0.280
General PTV, D_98%_ (Gy)	60.00 (0.00)	58.84 (1.01)	0.001^f^
General PTV, D_2%_ (Gy)	63.38 (1.35)	63.36 (1.53)	0.649
General PTV, CI	0.85 (0.05)	0.86 (0.05)	0.233
General PTV, HI	0.05 (0.02)	0.07 (0.03)	0.002^f^
BSPTV, D_98%_ (Gy)	60.53 (0.50)	60.00 (0.00)	0.003^f^, 0.003^c,f^, 0.001^d,f^
BSPTV, D_2%_ (Gy)	63.48 (1.41)	63.36 (1.51)	0.975
BSPTV, CI	0.79 (0.07)	0.84 (0.05)	0.003^f^, 0.281^e^
BSPTV, HI	0.05 (0.02)	0.05 (0.02)	0.100
Ring PTV, D_mean_ (Gy)	40.27 (6.83)	38.89 (7.13)	0.001^f^
Ring BSPTV, D_mean_ (Gy)	40.68 (6.72)	39.43 (7.08)	0.001^f^
Ring PTV, volume (cm^3^)	195.61 (203.99)		0.001^b,f^
Ring BSPTV, volume (cm^3^)	201.21 (210.89)		
Affected lung, V20	16.69 (11.32)	16.20 (10.85)	0.005^f^
Contralateral lung, V20	0.36 (0.01)	0.36 (0.01)	1.000
Total lungs, V20	7.25 (6.40)	7.02 (6.00)	0.008^f^
Affected lung, V5	29.22 (14.87)	28.75 (14.43)	0.013^f^
Contralateral lung, V5	6.91 (11.71)	6.71 (11.56)	0.028^f^
Total lungs, V5	16.26 (12.72)	15.92 (12.37)	0.015^f^
Affected lung, D_mean_ (Gy)	9.18 (5.51)	8.93 (5.28)	0.005^f^
Contralateral lung, D_mean_ (Gy)	0.97 (1.43)	0.95 (1.42)	0.012^f^
Total lungs, D_mean_ (Gy)	4.79 (3.33)	4.67 (3.18)	0.003^f^
Spinal cord, D_1%_ (Gy)	16.14 (16.50)	15.72 (15.89)	0.009^f^

*HI* homogeneity index, *CI* conformity index

^a^Comparison of general PTV optimization plan with BSPTV optimization plan

^b^Comparison of volumes of ring PTV and ring BSPTV

^c^Comparison of D_98%_ of general PTV and BSPTV in the same general PTV optimization plan

^d^Comparison of D_98%_ of general PTV and BSPTV in the same BSPTV optimization plan

^e^Comparison of CI of general PTV in the general PTV optimization plan and CI of BSPTV in the BSPTV optimization plan

^f^*P* < 0.05

The volume of the Ring PTV was smaller than the volume of the Ring BSPTV (*p* = 0.001). The BSPTV plans had significantly lower mean dose for Ring PTV and Ring BSPTV compared to the general PTV plan (*p* = 0.001 and *p* = 0.001). Furthermore, compared with the general PTV plans, the BSPTV plans showed a decrease in the V5 and mean dose of the affected lung (*p* = 0.013, 0.005), contralateral lung (*p* = 0.028, 0.012), and total lungs (*p* = 0.015, 0.003) and a decrease in the V20 of the affected lung (*p* = 0.005) and total lungs (*p* = 0.008).

### Robust analysis

Table 5 shows the results of the robust analysis for both general PTV plans and BSPTV plans. The ratios of scenarios showed that 100% of the CTV was still above 100% or 95% (clinical specification) of the prescription dose calculated for both plans. Moreover, the results showed that the ratios of scenarios of the clinical specifications was not significantly different between the general PTV and BSPTV plans (mean value: 0.966 vs. 0.953, *p* = 0.317). For both general PTV and BSPTV plans, about 80% of the target coverage could still be achieved in all uncertainty scenarios.

**Table 5:** The ratios of scenarios satisfied the clinical specifications that the 100% target volume being above the 100% or 95% prescription dose for all patients

Patient nos.	General PTV plan (%)		BSPTV plan (%)	
	100% prescription dose	95% prescription dose	100% prescription dose	95% prescription dose
1	50	100	50	100
2	50	100	50	100
3	100	100	50	100
4	100	100	100	100
5	67	83	50	83
6	50	100	50	100
7	100	100	100	100
8	67	100	67	100
9	83	100	67	83
10	100	100	100	100
11	100	100	83	100
12	67	100	50	100
13	17	67	17	67
14	83	100	83	100
15	100	100	100	100

## Discussion

Compared to proton radiotherapy, photon radiotherapy physically cannot produce perfectly conforming dose distributions. There will be a presence of entrance and exit doses due to how photons interact with matter. In this study, we took advantage of the exponential relationship between the absorbed dose and radiological depth. Since small movements in a beam direction result in negligible dose deviation, we designed the BSPTV concept to spare normal tissues in the beam direction. Because of the different shapes of the target in different beam entrance directions, it is important to choose a suitable incidence angle of the beam according to the projection area values. However, surrounding OARs need to be considered when choosing the incidence angle of the beam. Moreover, the distance of the target to the isocenter of the field is related to the utilization efficiency of the radiation, therefore, choosing of the incident angle of the beam is a trade-off between different factors.

In a previous study, it was shown that suboptimal dose conformation could lead to a tighter margin for the target, because the prescription dose escaped out of the target [14]. In our phantom simulation, a better conformity target dose distribution was achieved in the hypothetical plans group (> 90%) compared to the 4-field IMRT plans group (> 80%). However, the plan robustness was not different between the two types of plans. Combined, these results suggested that the conformity of the dose distribution would not have much impact on the plan robustness when a high target conformity (> 80%) was achieved. In IMRT plans, a high target conformity (> 80%) could easily be achieved, and the plan robustness would not be subjected to the conformity of the dose distribution.

We chose the CTV projection area analysis to identify suitable beam angles to obtain a smaller BSPTV volume and sparing more surrounding lung tissues. The mathematical and geometrical analysis suggested that beams that irradiated perpendicular to the maximum average length of the target in the XOY plane obtained the minimum BSPTV volume. The sparing volume, which represents differences between the general PTV and BSPTV, is dependent on the accumulation of the target projection area in the YOZ plane. Especially for targets with an irregular shape, such as the example case in Fig. 3, the CTV projection area differences between the maximum and the minimum value could be 25% (Fig. 3a). The CTV projection area curve is symmetric for beam angles with 180° intervals. Suitable beam angles with a direction close to the tumor can be chosen according to the CTV projection area curve.

The value of the BSPTV is impacted by the beam angles and the number of beams. Table 3 shows that if the number of beams increased from 4 to 5, the volume differences between the general PTV and BSPTV reduced from 12.50 to 6.94%. The digit phantom simulation of plans with three, five, and seven coplanar beams also demonstrated that the volume differences between the general PTV and BSPTV reduced with an increasing number of beams. Taken together, these results suggested that the BSPTV concept is not suitable for treatment with a large number of beams, such as stereotactic body radiation therapy (SBRT), because the BSPTV might be the same with a general PTV.

The clinical specifications of BSPTV plans of cases 5, 9, and 13 could not be achieved (Table 5). The scenarios, which are not satisfied for the clinical specifications, are all scenarios with set-up uncertainties in SI directions. The reason for the underdosing might be that the target with set-up uncertainties in the SI directions are too close to the beam aperture, therefore, the lateral scattering dose may not be sufficient, especially in lung tissue.

Shusharina et al. [15] proposed a probabilistic clinical target distribution concepts implemented into the probabilistic optimization process in the head and neck cases. This allowed physicists and physicians to identify the most suitable trade-off between target coverage and sparing of surrounding normal tissues at the treatment planning stage, without having to modify or redraw a CTV. In addition, Witte et al. [16] also tested the probabilistic planning margin volume concepts in the simulated phantom. Watkins et al. [17] defined the definite target volume to deliver extremely high doses to sub-volumes of PTVs in multiple treatment sites. These three studies focused on the influence of the probabilistic dose distribution to the target coverage, and sparing of surrounding normal tissues is another strategy comparing to the BSPTV methods. Since the BSPTV strategy could be applied without modification of the optimization engine of the commercial treatment planning system, it is a much easier and more straightforward strategy.

A considerable amount of work remains to continue to be performed to further improve BSPTV optimization. Both the van Herk’s margin concept and our beam specific margin concept share the following assumptions: the treatment uncertainties follow a Gaussian distribution, which is not appropriate for SBRT treatment, and only rigid translations of the target were accounted for [4]. We will focus on calculating the effects of intra-fraction motion and inter-fractional uncertainties as well as analyzing the rotation and deformation uncertainties of the targets. And we will continue study the BSPTV of two or more targets in one patient, such as primary sites and mediastinal lymph nodes for lung cancer cases.

## Conclusion

Lung tumors are surrounded by normal tissues and OARs, and the low dose delivery to normal tissues needs to be limited. Hence, the IMRT plans with 3–5 beams could effectively control the low dose area in lung treatment. Thus, using BSPTV is highly suitable for the IMRT is plan strategy.

## Figures and Tables

**Fig. 4:**
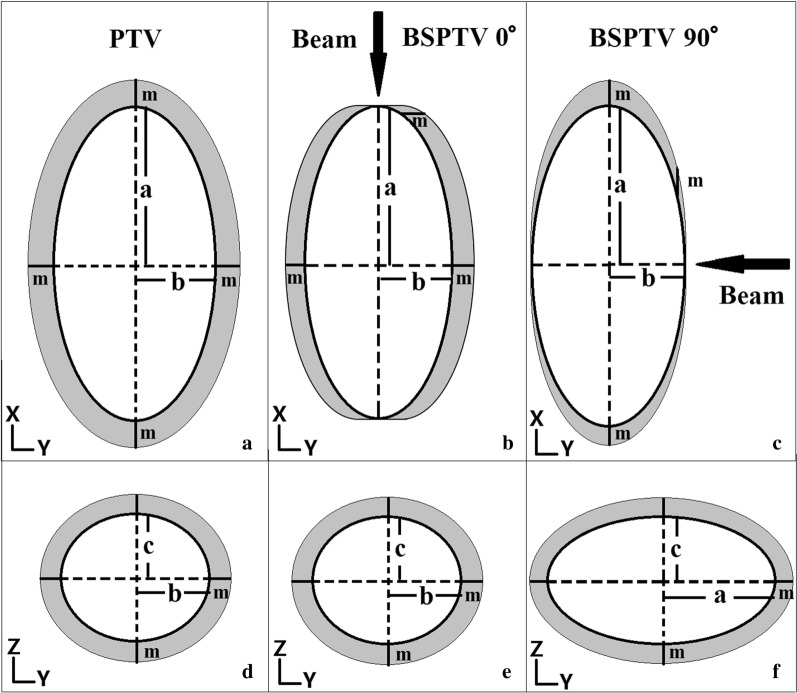
The projection area of the ellipsoid CTV and the general PTV in the XOY plane and YOZ plane are shown in **a**, **c**. The CTV (thick black line) is margined isotropically with *m* (gray area) in three directions to PTV (thin black line). The projection areas of the ellipsoid CTV and BSPTV of beam 0° in the XOY plane and YOZ plane are shown in **b**, **d**. The margin area (gray area) of BSPTV of beam 0° is smaller than that of the general PTV. The projection areas of the ellipsoid CTV and BSPTV of beam 90° in the XOY plane and YOZ plane are shown in **c**, **e**. The margin area (gray area) of BSPTV of beam 90° is smaller than that of both the general PTV and BSPTV of beam 0°

## Data Availability

Please contact author for data requests.

## Appendix

The relationship of the margin volume and the projection area of the target.

The total projection areas (S_YOZ_) of the target volume in a beam direction from 0° to 359° were calculated. In this research, CTV is the target. The margin volume of the convention PTV margin recipe will be:

1 $$V_{PTV} = \int_{0}^{{{\text{x}}_{0} + 2m}} \int_{0}^{{{\text{y}}_{0} + 2m}} \int_{0}^{{{\text{z}}_{0} + 2m}} dxdydz$$

where m is the length of the margin, *V*_*PTV*_ is the volume of PTV.

The margin volume of the BSPTV margin recipe will be:

2 $$V_{BSPTV} = \int_{0}^{{{\text{x}}_{0} }} \int_{0}^{{{\text{y}}_{0} + 2m}} \int_{0}^{{{\text{z}}_{0} + 2m}} dxdydz$$

where m is the length of the margin, *V*_*BSPTV*_ is the volume of BSPTV.

The volume differences between the two margin recipes are as following:

3 $$\Delta V = V_{PTV} - V_{BSPTV} = \int_{0}^{{{\text{x}}_{0} + 2m}} \int_{0}^{{{\text{y}}_{0} + 2m}} \int_{0}^{{{\text{z}}_{0} + 2m}} dxdydz - \int_{0}^{{{\text{x}}_{0} }} \int_{0}^{{{\text{y}}_{0} + 2m}} \int_{0}^{{{\text{z}}_{0} + 2m}} dxdydz = \int_{0}^{2m} \int_{0}^{{{\text{y}}_{0} + 2m}} \int_{0}^{{{\text{z}}_{0} + 2m}} dxdydz$$

*ΔV* is the volume differences between the two margin recipes.

If we need to spare more normal tissues, the *ΔV* needs to be the maximum. Hence, $\int_{0}^{{{\text{y}}_{0} + 2m}} \int_{0}^{{{\text{z}}_{0} + 2m}} dydz$ needs to be the maximum. Since the $\int_{0}^{{{\text{y}}_{0} + 2m}} \int_{0}^{{{\text{z}}_{0} + 2m}} dydz$ is in direct proportion to S_YOZ,_ the beam angle for the maximum of S_YOZ_ might be chose as the best beam angle to sparing the surrounding normal tissues.

Without loss of generality, we took the target as an ellipsoid with the lengths of axis (a (X axis) > b (Y axis) > c (Z axis)) as shown in Fig. 4 for example. The margin volume of the ellipsoid using the convention PTV margin recipe will be:

4 $$V_{PTV - ellipsoid} = \int_{0}^{{{\text{x}}_{0} + 2m}} \int_{0}^{{{\text{y}}_{0} + 2m}} \int_{0}^{{{\text{z}}_{0} + 2m}} dxdydz = \frac{{4}}{{3}}\pi (a + 2m) \cdot (b + 2m) \cdot (c + 2m)$$

where m is the length of the margin, *V*_*PTV-ellipsoid*_ is the margin volume of the ellipsoid using the convention PTV margin recipe

5 $$V_{BSPTV - ellipsoid} = \int_{0}^{{{\text{x}}_{0} }} \int_{0}^{{{\text{y}}_{0} + 2m}} \int_{0}^{{{\text{z}}_{0} + 2m}} dxdydz = \frac{{4}}{{3}}\pi (c + 2m)\int_{0}^{{{\text{x}}_{0} }} \int_{0}^{{{\text{y}}_{0} + 2m}} dxdy$$

where m is the length of the margin, *V*_*BSPTV-ellipsoid*_ is the margin volume of the ellipsoid using the BSPTV margin recipe. The maximum of *V*_*BSPTV-ellipsoid*_ is $\frac{{4}}{{3}}\pi a \cdot (b + 2m) \cdot (c + 2m)$ with the beam angle paraell to the X axis. The minimum of *V*_*BSPTV-ellipsoid*_ is $\frac{{4}}{{3}}\pi (a + 2m) \cdot b \cdot (c + 2m)$ with the beam angle paraell to the Y axis.

The volume differences between the two margin recipes are as following:

6 $$\Delta V_{ellipsoid} = V_{PTV - ellipsoid} - V_{BSPTV - ellipsoid} = 2m \cdot \int_{0}^{{{\text{y}}_{0} + 2m}} \int_{0}^{{{\text{z}}_{0} + 2m}} dydz = 2m \cdot S_{YOZ(m\arg in)}$$

*ΔV*_*ellipsoid*_ is the volume differences between the two margin recipes.

In order to obtain the maximum *ΔV*_*ellipsoid,*_ the S_YOZ_ needs to be the maximum as following:

7 $$S_{YOZ(m\arg in)} \mathop = \limits^{\Delta \max } \pi (a + 2m) \cdot (c + 2m)$$

8 $$\Delta V_{ellipsoid} = V_{ellipsoid} - V_{BSellipsoid} = 2m \cdot \int_{0}^{{{\text{y}}_{0} + 2m}} \int_{0}^{{{\text{z}}_{0} + 2m}} dydz = 2m \cdot S_{YOZ(m\arg in)} \mathop = \limits^{\Delta \max } \frac{8}{3}m\pi (a + 2m) \cdot (c + 2m)$$

where the beam angle for the maximum of S_YOZ_ is parallel to the Y axis and perpendicular to the X axis, the *ΔV*_*ellipsoid*_ is maximum and the *V*_*BSellipsoid*_ is the smallest. As shown in Fig. 4c, the beam angle for the maximum of S_YOZ_ is 90° in this ellipsoid case, and the beam angle for the mimmum of S_YOZ_ is 0° in this ellipsoid case.

Generalize to the general case, the value of S_YOZ_ is calculated as the product of the average length of target in the Z axis and the average length of target in the XOY plane. Since the beam is irradiated perpendicular to the Z axis, the average length of target in the Z axis is relatively a constant. Hence, the average length of target in the XOY plane will determine the value of S_YOZ_. The smallest volume of BSPTV is obtained with the beam irradiated perpendicular to the maximum average length of the target in the XOY plane.

## Abbreviations

CTV: Clinical target volume
PTV: Planning target volume
BSPTV: Beam-specific PTV
IMRT: Intensity modulated radiation therapy
OAR: Organ at risk
DVH: Dose volume histogram
SBRT: Stereotactic body radiation therapy
GTV: Gross tumor volume
SD: Standard deviation
